# Human testicular peritubular cells secrete pigment epithelium-derived factor (PEDF), which may be responsible for the avascularity of the seminiferous tubules

**DOI:** 10.1038/srep12820

**Published:** 2015-09-03

**Authors:** S. Windschüttl, C. Kampfer, C. Mayer, F. Flenkenthaler, T. Fröhlich, J. U. Schwarzer, F. M. Köhn, H. Urbanski, G. J. Arnold, A. Mayerhofer

**Affiliations:** 1 Biomedical Center (BMC), Cell Biology, Anatomy III, LMU, Munich, Germany; 2 Laboratory for Functional Genome Analysis (LAFUGA), Gene Center, LMU, Munich, Germany; 3 Andrologie-Centrum-München, Lortzingstraße 26, 81241, Munich, Germany; 4 Andrologicum Burgstraße 7, 80331, Münich, Germany; 5 Oregon National Primate Research Center, Oregon Health and Science University, Beaverton, Oregon, USA

## Abstract

Male fertility depends on spermatogenesis, which takes place in the seminiferous tubules of the testis. This compartment is devoid of blood vessels, which are however found in the wall of the seminiferous tubules. Our proteomic study using cultured human testicular peritubular cells (HTPCs) i.e. the cells, which form this wall, revealed that they constitutively secrete pigment epithelium-derived factor, PEDF, which is known to exert anti-angiogenic actions. Immunohistochemistry supports its presence *in vivo*, in the human tubular wall. Co-culture studies and analysis of cell migration patterns showed that human endothelial cells (HUVECs) are repulsed by HTPCs. The factor involved is likely PEDF, as a PEDF-antiserum blocked the repulsing action. Thus testicular peritubular cells, via PEDF, may prevent vascularization of human seminiferous tubules. Dihydrotestosterone (DHT) increased PEDF (qPCR) in HTPCs, however PEDF expression in the testis of a non-human primate occurs before puberty. Thus PEDF could be involved in the establishment of the avascular nature of seminiferous tubules and after puberty androgens may further reinforce this feature. Testicular microvessels and blood flow are known to contribute to the spermatogonial stem cell niche. Hence HTPCs via control of testicular microvessels may contribute to the regulation of spermatogonial stem cells, as well.

The capillary network in the human testis is complex. Capillaries are found between interstitial Leydig cells and within the several cellular layers of the wall of seminiferous tubules^1^,^2^. The latter run either semi-circumferentially around the seminiferous tubules or penetrate the peritubular compartment of the neighboring tubules. After leaving the wall of seminiferous tubules capillaries form a further capillary network. The capillaries, however, do not penetrate the basal lamina, on which Sertoli cells and spermatogonial cells reside on one side and the peritubular myoid cells on the other.

Neither the sex chords, during testis formation, nor the seminiferous tubules possess blood vessels and thus the avascular nature of the tubules precedes the onset of a well-defined blood-testis barrier. The latter is of outmost importance for spermatogenesis and comes into existence around puberty, when Sertoli cells form tight junctions and block any contact between the immune system and developing germ cells^3^. Yet, the proximity to blood vessels is of importance for spermatogonial stem cells. A study in mouse showed that the fate of spermatogonial stem cells depends on nearby blood vessels, which in case of the rodent are located in the interstitial space^4^. Rodents possess only one layer of peritubular cells and thus lack true peritubular capillaries, as seen in man, due to the multi-layered architecture of the tubular wall.

The capillary network is grossly changed in testicular cancer^5^ and it was suggested that peritubular cells, via their secreted products are involved in tumour growth, invasion and metastasis.

The peritubular cells of the human tubular wall can now be cultured^6^ and studied. They secrete a number of important factors, including glial cell line-derived neurotrophic factor (GDNF), which has a role in spermatogonial stem cell renewal. A recent proteomic analysis revealed the full spectrum of secreted factors. Among them are well known angiogenic factors, including, angiopoietin-related protein 2 (ANGPTL2) and vascular endothelial growth factor C, (VEGF-C), as well as the anti-angiogenic factor pigment epithelium-derived factor, PEDF^7^. To our knowledge expression of PEDF in the testis has not yet been reported.

PEDF is a multifunctional protein, which has among others a potent role in suppressing vascularization^8^. In endothelial cells PEDF was reported to promote apoptosis in new remodelled vessels^9^ and it has been reported to exert anti-tumor properties. Konson and co-workers reported pro-apoptotic actions of PEDF in human umbilical vein endothelial cells (HUVECs)^10^. At the molecular level, the mechanisms of PEDF-actions are not well understood. This is related to the fact that several binding partners are reported for PEDF. PEDF binds with high affinity to a lipase-linked membrane protein, namely the Patatin-Like Phospholipase Domain-Containing Protein 2 (PNPLA2), which therefore is regarded as the main receptor for PEDF.

Seminiferous tubules of the testis are avascular. The reasons for this fact are not known. Since a previous study^7^ revealed that HTPCs secrete pro-angiogenic factors and the anti-angiogenic factor, PEDF we aimed to explore the net-effect of HTPC-derived factors on human endothelial cells. To this end we studied the interaction of HTPCs with HUVECs. We also studied expression of PEDF in the immature testes of a nonhuman primate, and possible regulation of pro-/anti-angiogenic factors by androgens in HTPCs. The results revealed that HUVECs are repulsed by HTPCs, in a PEDF-dependent manner, that PEDF is expressed before puberty and that its levels are increased by androgens. Thus the cells of the tubular wall may control testicular blood vessels.

## Results

**Figure 1:**
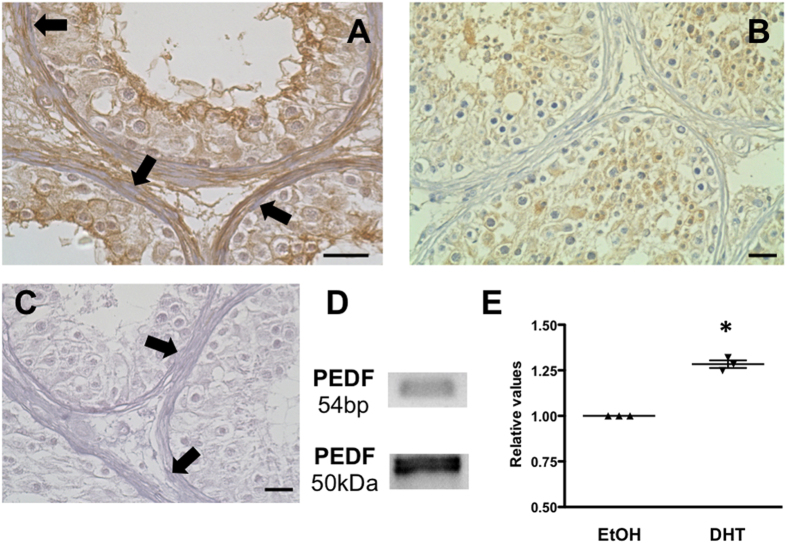
HTPCs secrete PEDF *in vivo* and *in vitro* and PEDF is regulated by DHT. (**A**) Immunohistochemistry using a human testicular biopsy reveals PEDF in the peritubular compartment, in cells and extracellular matrix (arrows point at peritubular cells). Hematoxylin counterstain of cell nuclei. (**B**) Preadsorption control and (**C**) negative control (goat normal serum instead of first antibody); each bar represents 10 μm. Hematoxylin counterstain of cell nuclei. (**D**) RT-PCR and Western Blot experiments confirmed PEDF expression in HTPCs. (**E**) Summary of qPCR studies showing significantly increased mRNA levels of PEDF when HTPCs were treated with 10 μM DHT for 3d (n = 3). Dot plots depict the individual levels, which are normalized to the solvent ethanol, based on calculated Ct values of treatment and the housekeeping gene L19. p < 0.05 (*).

Following on our initial description of PEDF as a secreted factor of HTPCs^7^, which stem from men with normal spermatogenesis, we verified its presence in additional five HTPC samples stemming now from men with impaired spermatogenesis (not shown). PEDF was readily detected as an abundantly secreted protein employing nano-LC-MS/MS, as described^7^. Using a specific antibody, we found immunoreactive, pre-adsorbable PEDF in human testicular biopsy tissue. It was in particular associated with the extracellular matrix and the cells of the tubular wall (Fig. 1).

Peritubular cells of the testis are known to express androgen receptors^2^ and HTPCs retain this expression. To activate androgen receptors we used dihydrotestosterone (DHT), which cannot be aromatized. DHT treatment for 3 days slightly but significantly increased the mRNA levels of PEDF, implying a hormonal control of this factor. We also found a small but significant decrease of mRNA levels of pro-angiogenic factors (VEGF-C and ANGPTL2) after DHT treatment (Supplementary data). Analysis of gene expression in infantile rhesus monkey testes (n = 3) showed that PEDF and its receptor are readily found already prior to puberty, indicating that other factors than androgens govern its expression, as well (Supplementary data).

**Figure 2:**
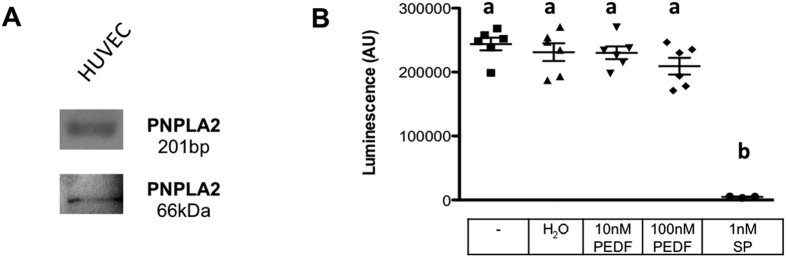
HUVECs express PNPLA2 and recombinant human PEDF does not influence viability. (**A**) RT-PCR analysis revealed the presence of a PEDF receptor (PNPLA2) mRNA and a specific PNPLA2 antibody detected PNPLA2 protein expression in HUVECs. (**B**) The results of a viability assay based on the levels of ATP indicate that PEDF (10 nM and 100 nM) does not influence the ATP concentration within 24 h, whereas the positive control, 1 nM Staurosporin (SP) significantly reduced viability. Number of replicates is n = 6. One-way ANOVA, *p* < 0.05 (a versus b). Bars are means +/− SEM.

To examine a vascular role of HTPC-derived PEDF, we turned to HUVECs. HUVECs express the receptor molecule for PEDF, PNPLA2, as revealed by RT-PCR and Western blotting. A recombinant PEDF peptide did not affect viability of HUVECs, indicated by unchanged levels of ATP (Fig. 2).

**Figure 3:**
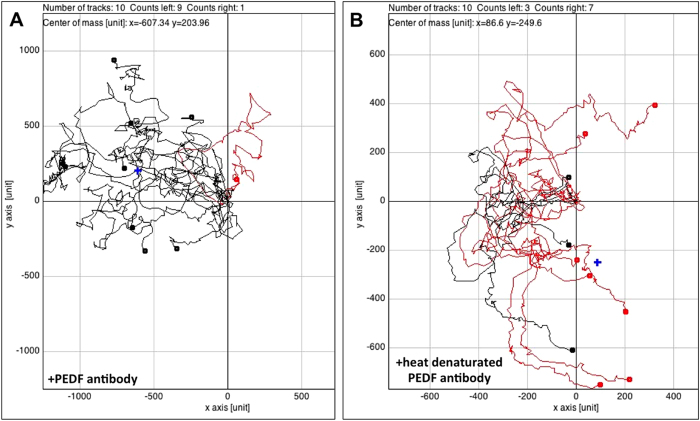
HTPCs via secreted PEDF influence the migration behaviour of HUVECs. Time-lapse movies (see Supplement 2 for movies) covering a 48 h period of co-culture (HTPCs and HUVECs) experiments were recorded and corresponding migration plots were generated (**A,B**). Experiments were repeated three times. The migration behaviour of randomly chosen HUVECs (n = 10) were tracked and are indicated by colour marks. Lines show migration tracks and endpoints (dots). Red tracks indicate cells, which migrated to the right and black tracks indicate cells migrating to the left hand side, i.e. towards the gap separating HUVECs and HTPCs at time of seeding. A blue cross symbolizes the centre of mass (corresponding to the area occupied by the majority of cells) in each migration plot. (**A**) The analysis of a co-culture experiment with HTPCs and HUVECs incubated in media containing 1.5 μg/ml of a specific antibody recognizing PEDF showed a directional movement of 9 tracked HUVECs towards the gap and the HTPCs. One single cell ends its track on the right hand side (red line). The centre of mass is thus shifted to the left hand side (x = −607.34; y = 203.96). (**B**) Analysis of an experiment, in which 1.5 μg/ml of the same PEDF antibody as in A was present. However, it was heat-inactivated by boiling for 30 minutes at 95 °C. The endpoints of 7 HUVECs are located to the right and 3 to the left. The centre of mass is close to the starting point (x = 86.6; y = −249.6).

We employed co-culture of HUVECs and HTPCs and imaged cellular behaviour (Fig. 3 and Supplementary data). We observed that HTPCs repulsed HUVECs and limit their expansion in cell culture. Individual cell tracking revealed that repulsion was initiated when the two different cell types approached each up to several μm. Thus repulsion appeared to be independent of direct cell-cell contact between HTPCs and HUVECs. Addition of a PEDF antiserum reduced this action, heat-inactivation of the antiserum reverted the effect. Thus, although HTPCs secrete known angiogenic factors^7^, the actions of the PEDF predominate.

## Discussion

Testicular blood vessels do not penetrate the basal lamina that separates Sertoli cell/basal germ cells from the peritubular cells and the absence of blood vessels is observed as early as in sex cords of the developing male gonad. The tubular compartment i.e. the seminiferous tubules of the adult human testis are therefore devoid of blood vessels. What factors are responsible for the development and the maintenance of the avascular nature of seminiferous tubules was not known.

The avascular nature of seminiferous tubules is complemented by the blood testis barrier (BTB), which is formed by tight junctions between Sertoli cells^3^. It effectively blocks any uncontrolled access of blood-born molecules to the tubular compartment, in which spermatogenesis occurs. Breakdown of the BTB is deleterious for spermatogenesis, and hence the formation of the functional BTB, which is an androgen dependent event, is initiated when spermatogenesis starts, i.e. around puberty^3^. Avascularity of the tubular compartment and a functional BTB are crucial for orderly spermatogenesis.

Supplying oxygen and nutrients to the cells of the tubular compartment is nevertheless crucial and was further implicated in the regulation of the spermatogonial stem cell niche^4^. The testicular capillaries involved are restricted to the interstitial space of testis of all species, but in human they are also found within the several cellular layers of the wall of the seminiferous tubules^1^. HTPCs are the cells of the testicular peritubular wall, which as we found, express the known anti-angiogenic protein PEDF. Like in a previous study^7^, analysis of five further samples indicated that it represents a highly abundant, secreted factor. Additionally, pro-angiogenic proteins were found. Studies about neo-vascularization and homeostasis in divers tissues report on the importance of a defined balance between both groups of factors^11^,^12^,^13^.

In this study we identified and confirmed PEDF as a factor derived from peritubular cells of the human testis *in vitro* and *in vivo* and identified its ability to interfere with human endothelial cells. In a previous study cellular expression of PEDF entailed cell death of HUVECs^10^, yet in our study addition of recombinant human PEDF did not cause cell death in HUVECs, even at high concentrations. Rather, our co-culture studies with HTPCs and HUVECs indicate that HUVECs are repulsed by HTPCs. Secreted factors are likely responsible and we pinpointed secreted PEDF to exert this influence. This assumption is supported by the ability of a PEDF antiserum to block the repulsing actions.

High testicular levels of PEDF before puberty imply that this molecule may function already during development in a non-human primate species. The expression of PEDF by HTPCs was increased when DHT was added to the culture medium. This emphasizes the importance of androgens, not only for the initiation of spermatogenesis and for the development of the BTB, but also for the general avascularity of the seminiferous tubules. Leydig cells, the producers of androgens, are not homogeneously distributed within the testis. A local regulation of PEDF, in concert with production of pro-angiogenic factors, may consequently be responsible for the maintenance of the complex network of the capillaries of the human seminiferous tubules and thereby may contribute to the spermatogonial stem cell niche. In support for such a scenario we found that HTPC-derived factors linked to angiogenesis, VEGF-C and ANGPTL2, were also influenced by DHT, but in a negative way. Compared to PEDF, they appear to be much less abundant secretory factors of HTPCs^7^, hence their roles remain to be further studied. Androgens are able to stimulate pro-angiogenic factors in other tissues^14^,^15^ but this may not apply to HTCPs and their products VEGF-C and ANGPTL2. It remains to be studied whether PEDF, VEGF-C and ANGPTL2 have a role in testicular cancer^9^,^10^, in which the architecture of the testicular microvessels is strikingly altered.

In conclusion, the results of this study suggest that the avascularity of the seminiferous tubules of the human testis is likely due to the secretion of PEDF by peritubular cells. The data emphasize the importance of these inconspicuous cells for testicular functions.

## Material and Methods

### Cell culture and treatment

HTPCs were isolated as described elsewhere^16^,^17^. The project was approved by the local ethic committee (Ethikkommmission, Technische Universität München; project 3051/11) and all patients provided a written informed consent. The methods were carried out in accordance with the approved guidelines. HTPCs were derived from men with normal spermatogenesis and were in passage 6 to 13. Cells were cultured in Dulbecco’s Modified Eagle Medium (DMEM) high glucose + 10% foetal calf serum (FCS; both from GE Healthcare; Freiburg, Germany) + 1% penicillin/streptomycin (PAN-Biotech; Aidenbach, Germany) HUVECs were cultured in endothelial. Cell Growth Medium, containing a supplement mix equal to 2% FCS (both Promocell; Heidelberg, Germany) + 1% penicillin/streptomycin (Promocell). All cells were cultured under standard conditions (37 °C, 5% CO_2_, 95% humidity). HUVECs were treated with 10 nM and 100 nM human recombinant PEDF (from HEK 293 cells, BioVendor R&D, Heidelberg, Germany) in Endothelial Cell Growth Medium with 2% FCS + 1% penicillin/streptomycin for 24 h. For some studies HTPCs were incubated with 10 μM DHT (Sigma; Deisenhofen, Germany) or the corresponding amount of the solvent (ethanol) for 3d in DMEM high glucose + 10% FCS + 1% penicillin/streptomycin.

### Immunohistochemistry

Immunohistochemical studies were performed as published previously^17^,^18^ using sections of human testicular biopsies of patients with normal spermatogenesis. A polyclonal rabbit anti-human PEDF antibody (1:50 ( = 0.7 μg/ml,) Sigma-Prestige), a biotinylated anti-rabbit secondary antibody (1:2500; Vector Laboratories, Inc. Burlingame, CA; USA) an avidin–biotin complex peroxidase (ABC, Vector Laboratories) and DAB (Sigma) were used. As described^19^, slices were incubated with a preadsorbed antibody (using human recombinant PEDF). As a further control the antibody was replaced by non-immune serum or IgG. Hematoxylin counterstained the cell nuclei. Sections were examined with a Zeiss Axiovert microscope, an Insight Camera (18.2 Color Mosaik) and Spot advanced software 4.6 (both from SPOT Imaging Solutions, Sterling Heights, MI, USA).

### RT-PCR and qPCR

Total RNA from cultured HTPC, HUVEC cells was prepared as described^20^ using the QIAGEN RNeasy minikit. In brief, a total amount of 400 ng of RNA was subjected to reverse transcription, using random primers (15-mer) and SuperScript II Reverse Transcriptase, 200 U/μl (Invitrogen GmbH, Darmstadt, Germany). Intron-spanning primer pairs amplified specific products for PEDF (5´-CCATGATGTCGGACCCTAAG-3 and 5´-GAATGAACTCGGAGGTGAGG-3´) and for PNPLA2 (5´-GCTTCCTCGGCGTCTACTACGTCG-3´ and 5´-GCACCTTCAGCAGGAAACTGCGG-3´). PCR steps consisted of 35 cycles of denaturing (at 94 °C for 60 sec) annealing (at 58 °C for PEDF and 68 °C for PNPLA2 for 30 sec) and extension (at 72 °C for 60 sec). Sequencing of all PCR products verified their correct identity. qPCR studies were performed as published elsewhere^21^ using the QuantiFast SYBR Green PCR Master Mix 2x (Qiagen, Hilden, Germany) and 2 ng/μL cDNA and the same primer pair for PEDF as for RT-PCR. L19 served as housekeeper gene. Ct values were calculated by the comparative 2^−ΔΔCT^ method.

### Viability assay

CellTiter-Glo Luminescent Cell Viability Assay (Promega, Mannheim, Germany) detected the viability of HUVECs by determining the ATP level, which is proportional to the metabolic state of vital cells. 4000 cells/well were cultured over night in sextuples of a 96 well plate in medium +10% FCS. Two different concentrations of PEDF (10 nM and 100 nM) were added to the cells for 24 h in medium without FCS. Medium only and medium with the solvent for PEDF (H_2_0) served as negative control, 1 nM Staurosporine (Sigma) served as a positive control.

### Co-Culture live cell imaging

HTPCs and HUVECs were grown in their standard culture media over night in a culture dish (μ-Dish, Ø 35 mm, high; ibidi, Martinsried, Germany) separated by a chamber. The chamber was removed after 24 h and left a gap of 500 nm. The dish was filled with a maximum of 0.6 ml Endothelial Cell Growth Medium with 10% FCS + 1% penicillin/streptomycin. As control a PEDF antibody (1.5 μg/ml medium; Sigma-Prestige) was added, when culturing HTPCs and HUVECs together. Live cell observation for at least 48 h under incubator-like conditions was performed as described before^21^. Briefly, the optimal conditions (5% CO_2_, 37 °C, 95% relative humidity) were created by using a heating system and a gas incubation system for CO_2_ and O_2_ (both ibidi). Time-lapse series were generated by taking a picture every 20 minutes with a ProgRes MF camera (Jenoptik, Jena, Germany), the Micro-Manager 1.3 Microscopy Software (Ron Vale’s laboratory at UCSF, USA) and a transmitted light microscope (Axiovert 135; Zeiss). Migration movements of 10 HUVECs were visualized by using the ImageJ plugin “Manual Tracking” (Abrice Cordelieres, Institut Curie, Orsay, France). Migration plots according to the selected cells were created with the ImageJ plugin “Chemotaxis and Migration Tool” (Version 1.01; ibidi, Martinsried, Germany).

### Western Blotting

Immunoblotting with cell culture lysates of HTPCs and HUVECs was performed as published^22^ using a polyclonal rabbit antiserum against PEDF (1:1000; bioproducts, Basel, Switzerland) and a monoclonal mouse antibody against PNPLA2 (1:1000; abnova, Paderborn, Germany). The detection of Western blot bands was done with HRP-conjugated corresponding secondary antibodies and chemiluminescent solutions (SuperSignal® West Femto Maximum Sensitivity Substrate; Pierce, Thermo Scientific, Rockford, IL, USA).

### Statistical analyses

Data were analyzed using GraphPad Prism^®^ (Prism 4.0a; GraphPad Software, Inc., La Jolla, CA, USA) using one way ANOVA followed by Tukeys post hoc test or column statistics, one sample t-test. All values shown are mean +/− SEM.

## Additional Information

**How to cite this article**: Windschüttl, S. *et al.* Human testicular peritubular cells secrete pigment epithelium-derived factor (PEDF), which may be responsible for the avascularity of the seminiferous tubules. *Sci. Rep.* **5**, 12820; doi: 10.1038/srep12820 (2015).

## Supplementary Material

Supplementary Information

Supplementary Movie 1

Supplementary Movie 2
