# Supplementary material for: Human testicular peritubular cells secrete pigment epithelium-derived factor (PEDF), which may be responsible for the avascularity of the seminiferous tubules
Source: Sci Rep. 2015 Sep 3;5:12820. doi: 10.1038/srep12820 (PMC4986702; doi:10.1038/srep12820)
Supplement: Supplementary Information [file srep12820-s1.pdf]

Title Page for Supplementary Information

Human testicular peritubular cells secrete pigment epithelium-derived factor (PEDF), which may be responsible for the avascularity of the seminiferous tubules

S Windschüttl[1], C Kampfer [1], C Mayer [1], F Flenkenthaler [2], T Fröhlich [2], JU Schwarzer[3], FM Köhn[4], H Urbanski [5], GJ Arnold[2], A Mayerhofer[1*]

[1] Biomedical Center (BMC), Cell Biology, Anatomy III, LMU, Munich, Germany

[2] Laboratory for Functional Genome Analysis (LAFUGA), Gene Center, LMU, Munich, Germany

[3] Andrologie-Centrum-München, Munich, Germany

[4] Andrologicum, Munich, Germany

[5] Oregon National Primate Research Center, Oregon Health and Science University, Beaverton, Oregon, USA

Supplementary information

Supplementary data 1:

Relative gene expression levels of SERPINF1 (= PEDF) and PNPLA2 (= PEDF receptor) in juvenile monkey testis (for method and samples, see; Adam, M. *et al.* High levels of the extracellular matrix proteoglycan decorin are associated with inhibition of testicular function. *Int. J. Androl.* **35**, 550-561 (2012)).

Animal age	SERPINF1 (PEDF)	PNPLA2
1YR, 82 days	1112,3	377
2YR, 101days	1410,4	453,3
1YR, 47days	1293,1	516,8

Supplementary data 2:

Time-lapse experiments and migration plots corresponding to the tracks shown in Figure 3 A and B. Note that the corresponding movies are provides as separate files.

HTPCs – HUVECs:
48h (+ 1.5µg/ml PEDF antibody)

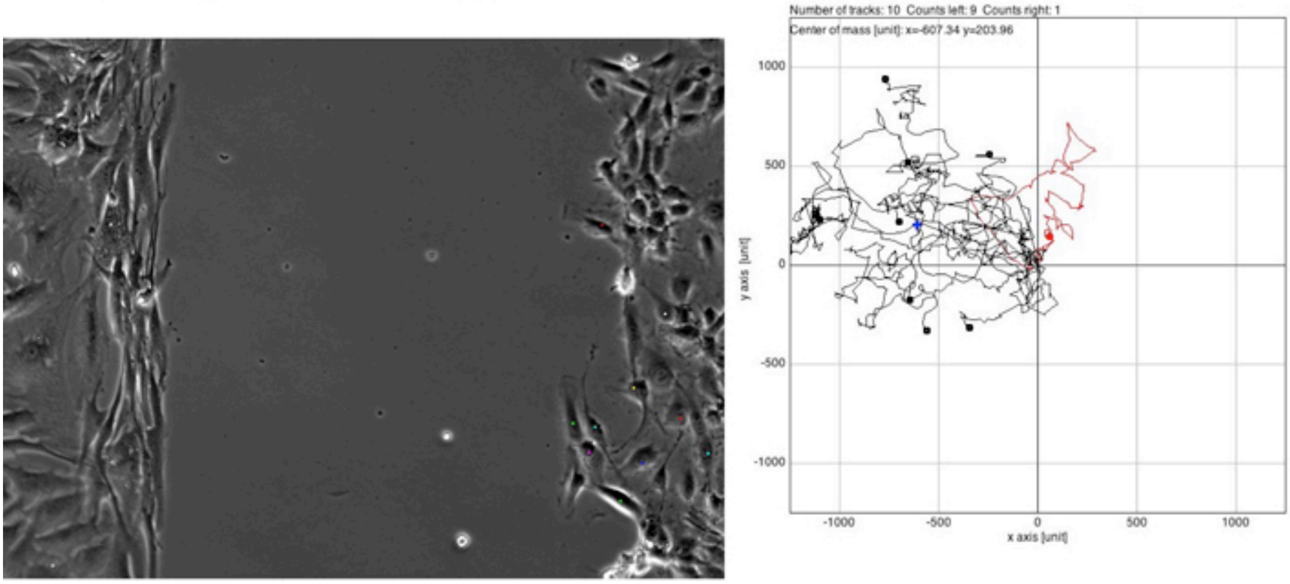

HTPCs – HUVECs:
48h (+ 1.5µg/ml heat-inactivated PEDF antibody)

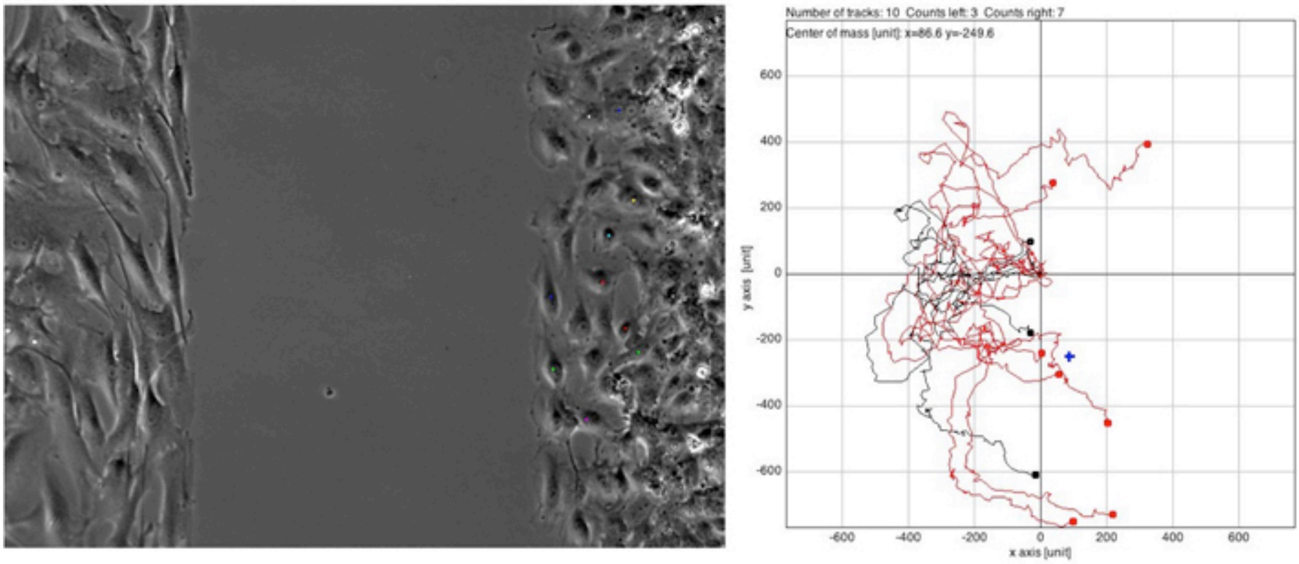

Supplementary data 3:

Summary of qPCR studies showing significantly but significantly decreased mRNA levels of VEGF-C (A) and ANGPTL2 (B) when HTPC were treated with 10µM DHT for 3d (n=3). Dot plots depict the individual levels, which are normalized to the solvent ethanol, based on calculated Ct values of treatment and the housekeeping gene L19. $p < 0.05$ (*).

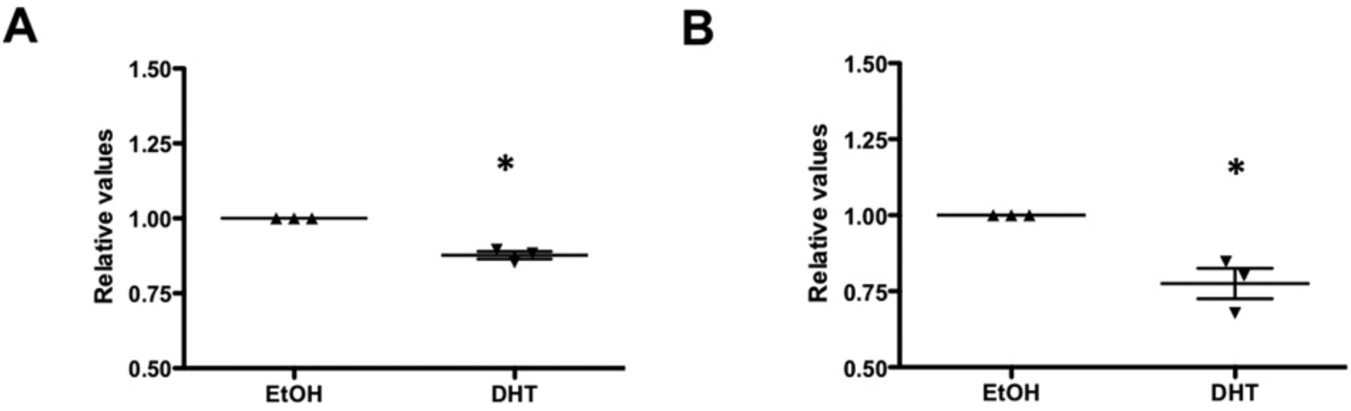

Supplementary data 4: Methods

LC-MS/MS analysis

The secretome of HTPC supernatant (24h in culture) of five individual donors (with impaired spermatogenesis) was observed with LC-MS/MS analysis as described before **(**Flenkenthaler, F. *et al.* Secretome analysis of testicular peritubular cells: a window into the human testicular microenvironment and the spermatogonial stem cell niche in man. *J. Proteome Res.* **13**, 1259-1269 (2014)).

Microarray analysis

This method was described previously (Adam, M. *et al.* High levels of the extracellular matrix proteoglycan decorin are associated with inhibition of testicular function. *Int. J. Androl.* **35**, 550-561 (2012)).Testicular samples from 3 juvenile monkeys were studied using Affymetrix GeneChip® microarray at the Oregon Health and Science University (OHSU), using the human HG_U133A GeneChip® microarray platform. Male rhesus macaques (*Macaca mulatta*) were cared for by the Division of Comparative Medicine at the Oregon National Primate Research Center (ONPRC), in accordance with the National Research Council's Guide for the Care and Use of Laboratory Animals, and were used in an Institutional Animal Care and Use Committee-approved study. The rhesus macaques had previously been involved in unrelated studies, and their post-mortem tissues became available through the ONPRC Tissue Distribution Program. The post-mortem collection of tissues involved sedating the animals with ketamine (15–25 mg/kg i.m.) and pentobarbital sodium (25–30 mg/kg i.v.) followed by exsanguination. This method of euthanasia is consistent with the recommendations of the American Veterinary Medical Association's Panel on Euthanasia.

RT-PCR and qPCR

Total RNA from cultured HTPC cells was prepared as described (Rey-Ares, V. *et al.*

Dopamine receptor repertoire of human granulosa cells. *Reprod. Biol. Endocrinol.* **5**, 40 (2007) using the QIAGEN RNeasy minikit. In summary a total amount of 400ng of RNA was subjected to reverse transcription, using random primers (15-mer) and SuperScript II Reverse Transcriptase, 200 U/µl (Invitrogen GmbH, Darmstadt, Germany). qPCR studies were performed as published elsewhere[21] using the QuantiFast SYBR Green PCR Master Mix 2x (Qiagen, Hilden, Germany) and 2 ng/µL cDNA. Intron-spanning primer pairs amplified specific products for VEGF-C (5´-ATTAGACGTTCCCTGCCAGC-3´ and 5´-TCTTCCTGAGCCAGGCATCT-3´) and for ANGPTL2 (5´-TGCGACCAGAGACACGAC-3 and 5´-CCGTCAATGTTCCCAAACCC-3´). L19 served as housekeeper gene and cDNA of EtOH treated cells as negative control. PCR steps consisted of 35 cycles of denaturing (at 94°C for 60 sec) annealing (at 60°C) and extension (at 72°C for 60 sec). Ct values were calculated by the comparative $2^{-\Delta\Delta CT}$ method.
